# Online Biomedical Resources for Malaria-Related Red Cell Disorders

**DOI:** 10.1002/humu.22330

**Published:** 2013-04-08

**Authors:** Frédéric B Piel, Rosalind E Howes, Oscar A Nyangiri, Catherine L Moyes, Thomas N Williams, David J Weatherall, Simon I Hay

**Affiliations:** 1 Spatial Ecology and Epidemiology Group, Tinbergen Building, Department of Zoology, University of OxfordSouth Parks Road, Oxford, United Kingdom; 2 Kenya Medical Research Institute/Wellcome Trust Programme, Centre for Geographic Medicine Research-Coast, Kilifi District HospitalKilifi, Kenya; 3 Nuffield Department of Clinical Medicine, University of OxfordOxford, United Kingdom; 4 Weatherall Institute of Molecular Medicine, University of OxfordOxford, United Kingdom

**Keywords:** G6PD, HbC, HbE, HbS, malaria, database, thalassemia

## Abstract

Warnings about the expected increase of the global public health burden of malaria-related red cell disorders are accruing. Past and present epidemiological data are necessary to track spatial and temporal changes in the frequencies of these genetic disorders. A number of open access biomedical databases including data on malaria-related red cell disorders have been launched over the last two decades. Here, we review the content of these databases, most of which focus on genetic diversity, and we describe a new epidemiological resource developed by the Malaria Atlas Project. To tackle upcoming public health challenges, the integration of epidemiological and genetic data is important. As many countries are considering implementing national screening programs, strategies to make such data more accessible are also needed.

## Introduction

Between the 19th century and the mid-1980s, epidemiology became a fast-evolving discipline aimed at the study of risk factors in human diseases and their spread. Clinicians and researchers rapidly became aware that, by gathering information on populations, diseases and the environment, they could improve knowledge of how to control, treat, eliminate, or even eradicate diseases and communicate this knowledge to the relevant authorities [Cerda and Valdivia, 2007; Whitelaw, 1923]. The recent surge of attention toward eliminating malaria illustrates the importance of epidemiology in reducing disease burden [Das and Horton, 2010]. Genetic epidemiology brings genetic factors into our understanding of disease risk and transmission. The spread of genetic disorders is driven by processes such as migration and selection over timescales of generations. These timescales make understanding these epidemiological features equally as important as those of rapidly transmitted infectious diseases because of their long-term burden, both at the individual and population level. Assembling epidemiological data about these disorders and implementing appropriate health policies is highly involved and may require population screening, genetic counseling, and antenatal diagnosis. A good illustration of the implementation of such interventions to prevent thalassemias is the example of Cyprus in the 1970s [Angastiniotis and Hadjiminas, 1981; Weatherall and Clegg, 2001].

Variation in the hemoglobin gene family is amongst the best-characterized genetic systems [Marks, 1989]. SCA (MIM #603903) was the first disease linked to the hemoglobin protein [Pauling et al., 1949] and genetically characterized [Ingram, 1956]. Many abnormal hemoglobin variants were discovered in the 1950s and 1960s because of the differences in their electrophoretic properties. Since the 1980s, DNA sequencing and genotyping studies have enabled confirmation of the large diversity of these variants, identification of many more new variants, and investigation of the underlying selection mechanisms driving this diversity.

Despite significant progress in our knowledge of malaria-related red cell disorders [Hedrick, 2011; Kwiatkowski, 2005], researchers, clinicians, and the public health community are currently confronted with important challenges. First, there is growing evidence that the global health burden of these inherited disorders is likely to increase [Weatherall, 2010]. Because of population growth and higher reproduction rates in areas of high frequencies for malaria-related red cell disorders, the incidence and prevalence of individuals affected globally are both expected to increase. This trend is reinforced in low- and middle-income countries by important reductions in child mortality resulting from significant improvements in hygiene, nutrition, and other public health interventions. The survival of children affected by genetic disorders who would have previously died undiagnosed would thus translate into a further increase in the incidence of these genetic disorders [Akinyanju, 2010; Balgir, 2000; Makani et al., 2007; Weatherall, 2011]. In high-income countries, growing fluxes of migrants from areas with high frequencies of these disorders results in increasing demands for screening programs, genetic counseling and healthcare services [Hassell, 2010; Streetly et al., 2009]. To quantify these spatial and temporal changes, it is important to gather detailed epidemiological information to assess the current and future burdens faced by countries. This is particularly needed in areas in which population surveys for these disorders are lacking, but also in areas where only out of date information is available. Second, these disorders present considerable geographical heterogeneity in terms of prevalence and genetic diversity, resulting in complex phenotype–genotype relationships, particularly for the thalassemias [Weatherall, 2001] and G6PD deficiency [Mason et al., 2007]. Individuals with similar genotypes, exposed to different environmental conditions, can present very different phenotypes. Furthermore, societal behaviors or customs (e.g., consanguinity) can also lead to higher frequencies of severe forms of a disease and the recent globalization of human migrations has led to the appearance of new compound statuses of different disorders, which would be highly unlikely to have occurred otherwise. To implement appropriate measures to reduce the clinical and economic burden associated with these disorders, it is crucial to improve our knowledge about these relationships. This relies on collecting reliable epidemiological and genetic data based on accurate diagnostics.

Until recently, most of the afore-mentioned disorders have not enjoyed a high priority on public health agendas, despite a relatively high impact on childhood mortality, particularly in Africa [Grosse et al., 2011]. Following decades of advocacy, the United Nations finally recognized hemoglobinopathies as a public health problem in 2006 [United Nations, 2008; World Health Organization, 2006]. These disorders have also been included in the Global Burden of Diseases, Injuries, and Risk Factors Study 2010 (GBD 2010, http://www.globalburden.org) as part of the Non-Communicable Diseases Cluster [Murray et al., 2012]. In parallel with this growing awareness, epidemiological evidence, supported by genetic studies, is increasingly needed by Ministries of Health and public health organizations to implement appropriate policies.

Here, we provide (1) a short description of seven malaria-related red cell disorders; (2) we review online global databases that include significant data on at least one of these disorders and we describe several challenges related to keeping these resources up to date and accessible; (3) we present a new open-access spatial database developed by the Malaria Atlas Project (MAP, http://www.map.ox.ac.uk) that currently holds data on four of these disorders and describes future work to expand this resource; and (4) we advocate the value of including spatial data in genetic databases to be used for epidemiological studies.

## Malaria-Related Red Cell Disorders

Many hundreds of genetic conditions affecting the red blood cells (RBCs) have been identified worldwide [Weatherall et al., 2006]; however, we concentrate here on those sufficiently common to be of public health significance. We have a particular interest in genetic variants that interact with the parasitic disease malaria because the public health importance of these variants extends from their own clinical impact to the effect they have on the burden of malaria within populations in endemic countries [Flint et al., 1998; Hill, 1987; Hedrick, 2011; Williams, 2006). We have recently investigated the spatial support for such a relationship by looking at the distribution of hemoglobin S (HbS) and malaria endemicity [Piel et al., 2010]. The particular disorders we have focused on include the following hemoglobin mutations, enzymopathies, and RBC surface loci: the main structural hemoglobin variants: HbS (MIM #141900.0243) [Piel et al., 2010; 2013b; Serjeant and Serjeant, 2001], hemoglobin C (HbC; MIM #141900.0038) [Piel et al., 2013a], hemoglobin E (HbE; MIM #141900.0071) [Flint et al., 1998; Makani et al., 2007; Williams et al., 2005], and the thalassemias: respectively α- (MIM #604131) and β-thalassemias (MIM #613985) [Weatherall and Clegg, 2001]; glucose-6-phosphate-dehydrogenase (G6PD; MIM #305900) deficiency [Cappellini and Fiorelli, 2008; Howes et al., 2012; Luzzatto and Notaro, 2001; Ruwende et al., 1995]; Southeast Asian ovalocytosis (MIM #109270.0002) [Kidson et al., 1981], and Duffy negativity (MIM #613665.0002) [Carter, 2003; Howes et al., 2011; Livingstone, 1984; Menard et al., 2010; Miller et al., 1976]. For descriptive simplicity, we have grouped all of these conditions under the malaria-related red cell disorders terminology. Many detailed reviews have described each of these disorders comprehensively (e.g., Carter and Mendis, 2002; Hedrick, 2011; Kwiatkowski, 2005; Weatherall et al., 2006] and we provide here a very brief summary of their genetics and epidemiology.

1. Sickle hemoglobin or HbS is a structural variant of normal adult hemoglobin (HbA) caused by an amino acid substitution at position 6 of the β-globin chain (HBB c.20A>T; p.Glu6-Val) and is inherited as a Mendelian trait. Carriers or heterozygotes (HbAS) are almost always asymptomatic. Homozygotes (HbSS) suffer from sickle cell anemia (SCA), which often leads to acute and chronic complications including vaso-occlusive crisis, acute chest crisis or hemolytic crisis [Serjeant and Serjeant, 2001]. Sickle hemoglobin was largely restricted to Africa, the Middle East and parts of India but nowadays it is also common in the Americas, the Caribbean and Europe following human diasporas [Piel et al., 2013b].
2. HbC is another structural variant of HbA caused by an amino acid substitution (HBB c.19G>A; p.Glu6Lys) occurring at the same position. HbAC carriers are asymptomatic. HbCC causes clinically mild hemolytic anemia, because of the reduced solubility of the RBCs, which can lead to crystal formation. HbC is mainly of clinical significance when inherited in combination with HbS (sickle-HbC disease), causing chronic hemolytic anemia and intermittent sickle cell crises, slightly less severe or frequent than in HbSS [Powars et al., 2002], and when co-inherited with β-thalassemia (HbC-β thalassemia), causing moderate hemolytic anemia with splenomegaly [Weatherall and Clegg, 2001]. Previously, HbC was prevalent only in Western Africa but carriers can now be found much more widely [Piel et al., 2013a].
3. HbE is a structural variant of normal hemoglobin (HBB c.79G>A; p.Glu26Lys) affecting the production rate of HbA. Heterozygotes with HbAE are asymptomatic, whereas homozygotes can present some mild clinical features similar to individuals with β-thalassemia trait. Globally, compound individuals with HbE and β-thalassemia represent the highest burden with a wide range of clinical severity. The most severely affected individuals are transfusion dependent [Weatherall and Clegg, 2001]. HbE reaches frequencies up to 60% in parts of Thailand, Laos, and Cambodia, and is highly prevalent in India, Sri Lanka and Malaysia [Vichinsky, 2007; Weatherall and Clegg, 2001].
4. The thalassemias affect the rate of production of either the α- or β-globin chains that form the subunits of adult hemoglobin, leading to α- and β-thalassemia, respectively [Weatherall and Clegg, 2001]. Thalassemias are caused by a large variety of mutations and deletions, causing severity proportional to the inability to synthesize globin chains. Although α- and β-thalassemias are both genotypically classified into minor, intermediate and major forms, there is a continuum of phenotypes ranging from asymptomatic to lethal. They were originally found across the “thalassemia belt,” which extends from the Mediterranean area through the Middle East and India, to Southeast Asia. They are now also commonly found in many other parts of the world [Weatherall and Clegg, 2001].
5. The Duffy blood group is characterized by Duffy antigens, which are expressed on the surface of RBCs. Repeated exposure to different blood types may trigger immunogenic transfusion reactions in recipients, though this is rare. The Duffy gene has two main variants, which express the Fy^a^ and Fy^b^ antigens [Livingstone, 1984]. These antigens differ by a single amino acid (Gly42Asp), encoded by alleles FY*A and FY*B. A null ‘erythrocyte silent’ (ES) phenotype, caused by a point substitution in the gene's promoter region prevents gene expression, encoding the FY*A^ES^ and FY*B^ES^ alleles, the former (FY*A^ES^) having only been occasionally reported [Kasehagen et al., 2007; Sellami et al., 2008]. The Duffy negative phenotype (most commonly encoded by FY*B^ES^/*B^ES^) was thought to be fully protective against *Plasmodium vivax* infection, as the parasite was found to depend on the Duffy antigen for RBC entry [Miller et al., 1976], but recent evidence of *P. vivax* infected-Duffy negative individuals has brought the universality of this relationship into question [Menard et al., 2010; Mercereau-Puijalon and Menard, 2010; Wurtz et al., 2011; Zimmerman et al., 2013]. The most prevalent Duffy allele globally is FY*A, which reaches high frequencies (>90%) across East Asia, whereas in sub-Saharan Africa, the predominant allele is the silent FY*B^ES^ variant, commonly reaching frequencies approaching 100% and encoding the Duffy negative phenotype [Howes et al., 2011].
6. Glucose-6-phosphate dehydrogenase (G6PD) deficiency is the most common human enzyme disorder [Cappellini and Fiorelli, 2008], found throughout malarious regions, with an estimated overall allele frequency of 8.0% (50% uncertainty interval: 7.4%–8.8%) across malaria endemic countries [Howes et al., 2012]. Mutations in the gene cause reduced enzyme activity, leaving RBCs vulnerable to oxidative stress. Although the condition is typically asymptomatic, severe acute hemolysis can be triggered by certain foods, infections, and drugs. One such drug is primaquine: the only drug currently licensed to clear the relapsing stages of *P. vivax* malaria from the liver [Howes et al., 2013]. G6PD deficiency is also a main cause of neonatal jaundice in some regions. A large number of mutations cause this condition, and these vary in their clinical characteristics from none to highly severe. The most clinically severe G6PD deficient variants are found across Asia. Although the variants common among sub-Saharan African populations are considered less severe, the high prevalence of the deficient phenotype in this region means that the public health risks associated with G6PD deficiency are also high in this region [Howes et al., 2012].
7. Southeast Asian ovalocytosis (SAO) is an elliptocytosis, a genetic defect affecting the structural and functional properties of RBCs [Liu et al., 1990]. Heterozygotes are totally asymptomatic, whereas homozygotes are not viable [Delaunay, 2007]. SAO is mostly found in the malarious regions of Southeast Asia and the western Pacific [Rosanas-Urgell et al., 2012].

## Existing Resources

Although a number of national and regional resources have also been launched (for example the Centre for Arab Genomic Studies Database (http://www.cags.org.ae/) or the Indian Genetic Disease Database (http://www.igdd.iicb.res.in/)), the present review focuses only on global databases. Table 1 provides an overview of key characteristics of each of the resources described here.

**Table 1:** Overview of the Characteristics of Existing Resources on Malaria-Related Red Cell Disorders (Termed IBDs) and of the New Resource Launched by the Malaria Atlas Project (MAP-IBD)

			Survey locations provided				IBDs included						
					
Resource	URL	Years covered	National	Subnational	Communlity	Geographic coordinates (lat/lon)	HbS	HbC	Duffy	G6PD	Thal	SAO	Prevalence data
l_ivingstone	/	1904–1985	Yes	No	No	No	Yes	Yes	Yes	Yes	Yes	Yes	Yes
HGHG	/	1949–1994	Yes	n/a	n/a		Yes	Yes	Yes	Yes	No	Yes	No
FIDD	http://medic.cardiff.ac.uk/fidd/	n/a	Yes	Variable	No	No	Yes	No	No	Yes	Yes	No	Very limited
HbVar	http://globin.bx.psu.edu/hbvar			No	No		Yes	Yes	No	No	Yes	No	No
FINDbase	http://www.findbase.orR	n/a	Yes	Variable	No	Yes	Yes	No	No	Yes	Yes	No	No
ALFRED	http://alfred.med.vale.edu			n/a	Yes		Yes	Yes	No	Yes	Yes	No	Very limited
G6PD deficiency database	http://www.bioinf.org.uk/g6pd/	1986–2000	No	No	No	No	No	No	No	Yes	No	No	No
G6PD MutDB	http://bminfor.tongji.edu.cn/mutdb	1988–2009	No	No	No	No	No	No	No	Yes	No	No	No
MAP-IBD	http://www.map.ox.ac.uk	1950–2011	Yes	n/a	Yes	Yes	Yes	Yes	Yes	Yes	No	No	Yes

As early as the 1960s, Frank B. Livingstone started assembling a global database of the frequencies of hemoglobin variants, thalassemias, glucose-6-phosphate dehydrogenase deficiency, G6PD variants, and ovalocytosis in human populations. His dedication led to the publications of updated versions in the 1970s [Livingstone, 1973] and 1980s [Livingstone, 1985], which still represent a unique source of information on the prevalence of malaria-related red cell disorder variants among different population groups. Although Livingstone's last database has recently been reprinted [Livingstone and Marks, 2009], the absence of an electronic version of his tables and the crude spatial information associated with the location of each population survey hinder contemporary use of his databases. In the 1990s, Modell and Darlison updated his work on hemoglobin variants and gathered additional data on the thalassemias from research reviews, country visits, and the former WHO Working Group on Haemoglobin Disorders into an almanac [Modell and Darlison, 2008]. A significant amount of data have nevertheless been published since this study was conducted.

The History and Geography of Human Genes (HGHG) [Cavalli-Sforza et al., 1994], aimed at reconstructing human population history, using a novel approach combining population genetics and geography. Although now twenty years outdated, this book represents an invaluable resource containing 76,676 human gene frequency estimates. Investigations of hemoglobin disorders were limited because of their interactions with malaria. The HGHG included maps of the global distribution of HbS, HbC, Duffy negativity and G6PD deficiency, but the input data and the methodology used are not fully described, making it almost impossible to use them for quantitative analysis. An online version of the HGHG, named the Human Population Genetics Database (HPGD), was temporarily available on the Human Population Genetics Laboratory's Website (http://hpgl.stanford.edu/index.html) but, at the time of writing, was no longer operational.

The Frequency of Inherited Disorders Database (FIDD, http://medic.cardiff.ac.uk/fidd/), launched in 1998, aims to provide a systematic literature search summary on the prevalence and incidence of human Mendelian disorders [Al-Jader et al., 2001]. Survey information and phenotype prevalence are provided but this database only includes limited data on hematological disorders imported from the Online Mendelian Inheritance in Man compendium (14 records for sickle cell, 41 for alpha-thalassemia, 50 for beta-thalassemia, and 20 for G6PD deficiency). Locations are usually national or occasionally subnational but precise coordinate for the surveys reported are not given.

With the development of genomics in the 1990s, databases started to focus more on the genetic diversity of the new variants being regularly discovered than on their epidemiology and frequencies. HbVar (http://globin.bx.psu.edu/hbvar), launched in 2001, is a relational database on the genomic sequence changes leading to human hemoglobin variants and types of thalassemia [Hardison et al., 2002; Giardine et al., 2007; 2011; Patrinos et al., 2004]. It provides extensive information for each variant and mutation, including a description of the variant and associated pathology, hematology, electrophoretic mobility, methods of isolation, stability information, ethnic background, structure studies, functional studies, and references, but gives no details of gene frequencies or survey locations.

The Frequency of Inherited Disorders Database (FINDbase, http://www.findbase.org, launched 2005) is an online repository of information on the frequency of mutations causing inherited disorders [van Baal et al., 2007]. Data on thalassemias and G6PD deficiency from HbVar are included. Some mutation data are spatially referenced to the national level and occasionally to individual cities. It is far from comprehensive containing 768 records for beta-thalassemia, 18 for G6PD deficiency and none for other malaria-related red cell disorders.

The ALlele FREquency Database (ALFRED, http://alfred.med.yale.edu), launched in 1999, is a resource of gene frequency data on human populations supported by the U.S. National Science Foundation [Cheung et al., 2000]. The website allows users to visualize existing data and submit new data. The site includes extensive unpublished data, but only a tiny fraction (<1.5%) is on malaria-related red cell disorders (2 and 7 populations for Hb A/S/C and G6PD deficiency, respectively). A text description of the population surveyed is given but no coordinates are assigned to the location.

Finally, we identified a couple of resources specific to G6PD deficiency: (1) the G6PD deficiency database created by Dr Andrew Martin's group at University College London (http://www.bioinf.org.uk/g6pd/index.html) provides information on mutations leading to deficiency but no geographical information is provided; and (2) G6PD MutDB (http://bminfor.tongji.edu.cn/mutdb/) links mutations to deficiency phenotypes [Zhao et al., 2010] and while information on ethnic backgrounds is included, geographical location is not.

None of the above databases provide geographical coordinates for the communities surveyed. They do all provide citations that allow users to go back to the original source for further information and they allow users to extract key information from each record but they do not allow users to download integrated sets of community-level survey data from multiple records for use in epidemiological analyses.

Alongside these resources, others focus on increasing awareness of malaria-related red cell disorders. First, the Accessible Publishing of Genetic Information (APoGI, http://www.chime.ucl.ac.uk/APoGI, launched 2000) provides information and education materials on hemoglobin gene variants to help healthcare professionals on hemoglobin disorders to provide accurate counseling. The development of this resource was funded by the Wellcome Trust. No data on gene frequencies are available.

Second, the eInfrastructure for Thalassaemia Research Network (Ithanet, http://www.ithanet.eu, launched 2006) is an electronic infrastructure for a thalassemia research network developed within the European Union. Ithanet initially focused on the European community to facilitate contacts between researchers and data sharing. All hemoglobinopathies have recently been included in an extension of the project. It provides a community portal for experts, organizations and networks on thalassemias and other hemoglobinopathies. No data on gene frequencies are available.

Third, the Global Burden of Diseases, Injuries, and Risk Factors Study 2010 (GBD 2010, http://www.healthmetricsandevaluation.org/gbd) currently represents the most comprehensive effort to produce complete and comparable estimates of the burden of diseases, injuries, and risk factors for the years 1990, 2005, and 2010 for 21 regions globally. Sickle cell, G6PD deficiency, and the thalassemias have been included in the leading causes and risks, based on deaths, years of live lost, years lost to disability, and disability-adjusted life years for 1990 and 2010.

## The MAP's Contribution: A New Database

**Fig. 1:**
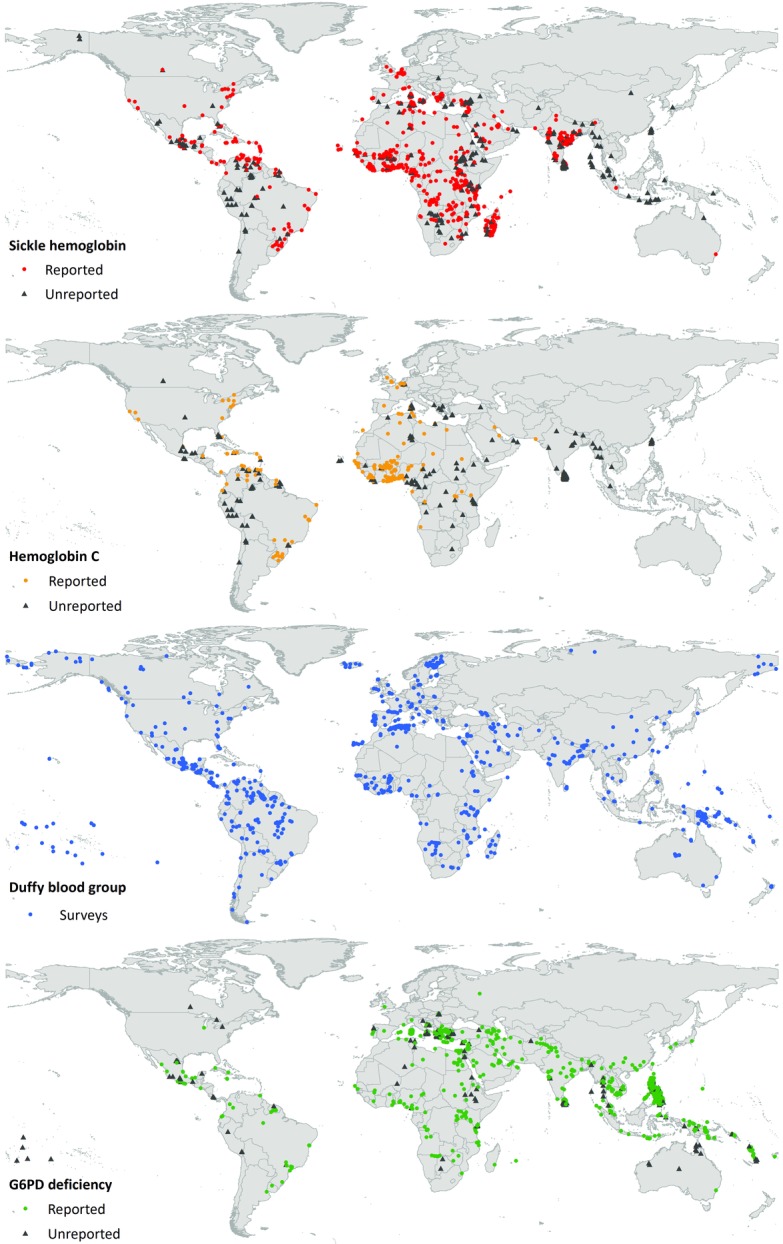
Global Maps of Surveys Included on Sickle Hemoglobin (HbS), Hemoglobin C (HbC), the Duffy Blood Group and G6PD Deficiency.

Although each of the resources described above represents invaluable sources of information, none of them provides sufficiently disaggregated information on the incidence and prevalence of malaria-related red cell disorders that could potentially be used by health policy makers in developing or targeting policies. Over the last four years, the MAP, which aims to disseminate free, accurate and up-to-date information on malaria and associated topics, organized on a geographical basis, has assembled data on the distribution and prevalence of selected malaria-related red cell disorders to create an open-access biomedical resource for researchers, clinicians and members of the public health community. This database was conceived to complement the existing online resources described above. Its main features include: (1) data based on detailed contemporary searches conducted across various online bibliographic databases, including Pubmed, ISI Web of Science and Scopus, as well as cross-referencing with existing databases (e.g., Livingstone 1985, HGHG 1994) and unpublished sources of data accessed through personal communications. Details of the protocols used have been previously published [Howes et al., 2012; 2011; Piel et al., 2010; 2013a; 2013b]; (2) surveys that are representative of local communities (i.e., excluding patient surveys or surveys targeting specific ethnic group(s), which risk being biased samples); and (3) geographic coordinates of all surveys, mapped to the highest precision possible to encapsulate spatial heterogeneity in the distribution and prevalence of these disorders (Fig. 1). Survey data from all sources can be downloaded as a single output ready for use in epidemiological analyses.

As summarized in Table 2, this database includes 1,211, 445, 922 and 665 data points for sickle hemoglobin, HbC, G6PD deficiency, and the Duffy blood group, respectively. Similar work on the thalassemias and HbE is ongoing. For sickle hemoglobin [Piel et al., 2010; 2013b], the data provided include the sample size and the number of individuals with the HbAA, HbAS, and HbSS genotypes. A subset of the data, corresponding only to representative surveys of autochthonous communities, reflects the original distribution of sickle hemoglobin, which was used to validate the malaria hypothesis spatially [Piel et al., 2010]. Similarly for HbC [Piel et al., 2013a], data are summarized as HbAA, HbAC and HbCC genotypes. For the Duffy blood group, five different categories of data are presented, corresponding to the type of diagnostic used: serological (includes three data types depending upon the antigens tested for) and molecular (two data types depending upon which loci were examined) [Howes et al., 2011]. National-level estimates of Duffy negative populations are also available. For G6PD deficiency, data are presented by sex, with the number of individuals tested and deficient at each survey site. Only phenotypically diagnosed samples are included in the database. These datasets for each disorder provide the density of population surveys and the observed spatial heterogeneity of each disorder. Survey publication date is also listed. This could be used, for example, to identify obvious gaps in the data currently available or to measure deviation from Hardy–Weinberg equilibrium. Protocols detailing the data collection and inclusion criteria are available for each disorder on the associated map pages in the “Browse Resources” section.

**Table 2:** Number of Data Sources, Spatially Unique Data Points and Individuals Tested for Each Malaria-Related Red Cell Disorder Included in the Database

Malaria-related red cell disorder	Data sources	Spatially unique data points released [*used in the model*]	Overall number of individuals tested
Sickle hemoglobin			
*Autochthonous populations*	278	730 [*773*]	347,434
*All populations*	435	1,129 [*1,211*]	9,032,377
Hemoglobin C	174	445 [*445*]	7,540,983
G6PD deficiency			
*Males*	254	910 [*1,720*]	316,448
*Females*	138	337 [*1,067*]	106,510
*Total*	258	922 [*1,734*]	422,958
Duffy blood group	301	665 [*821*]	114,563

Some of the surveys were included in the MAP modeling analyses, but authorization for public release was not granted. The figures in this table include all those freely available on the MAP Website.

The survey data described above form one part of a Web portal created to disseminate a range of data on malaria-related red cell disorders. As well as assembling these datasets, we have developed geostatistical models to make continuous predictions of the frequencies of each of these disorders in areas where no surveys were available [Diggle and Ribeiro Jr, 2007]. The models predicted continuous mapped surfaces of these disorders, as well as population estimates of affected individuals. All predictions are generated with associated uncertainty metrics [Patil et al., 2011]. Further information about these methodological aspects is available in the associated publications [Howes et al., 2011; 2012; Piel et al., 2010; 2013a; 2013b], linked from all of the MAP pages.

In addition to the raw survey data, we release model outputs in the form of map images (in PDF and PNG formats), GIS surfaces (in binary float and GeoTiff formats), and population estimates. These include continuous frequency maps of the distribution of sickle hemoglobin, HbC, Duffy negativity and G6PD deficiency, and their prediction uncertainty. For the first time, the Bayesian model-based geostatistical (MBG) approach used allowed us to account for the uncertainty associated with our predictions in a probabilistic way [Patil et al., 2010]. The maps presented show the mean or median predicted frequency and the interquartile range, which is a measure of uncertainty.

Such geographical maps can be used to support public health decisions through providing a spatially continuous estimate of the heterogeneity of the frequencies of these conditions. Furthermore, the maps permit estimates of individuals or newborns affected by these malaria-related red cell disorders to be made. Global, regional, national, and some subnational estimates in newborns are provided via the Web portal for sickle hemoglobin. Similar data are available for HbC in African countries. National population estimates are available for G6PD deficiency and Duffy negativity. The Duffy negativity map has been used to refine estimates of the population at risk of *P. vivax* malaria [Gething et al., 2012; 2010], whereas G6PD deficiency estimates provide information on areas in which primaquine therapy should be considered with caution [Howes et al., 2012].

Data searches can be performed for a given region (e.g., World Health Organization regions), country, topic and/or subtopic using the Resource Browser (http://www.map.ox.ac.uk/browse-resources/). Brief descriptions and links to complementary external resources, described above, are also available at http://www.map.ox.ac.uk/explore/inherited-blood-disorders/resources/. Individuals and organizations who have generously contributed unpublished data for use in the mapping models are listed at http://www.map.ox.ac.uk/acknowledgements/. Only data for which open-release permission was granted are included in the online database.

## The Ways Forward

The creation of this new resource by the MAP is a first step toward assembling a contemporary database of epidemiological data on malaria-related red cell disorders, alongside data on malaria parasites and vectors. During the data collection process, it became obvious that only a fraction of survey data is easily accessible in the public domain; much being unpublished or published in journals or reports with limited visibility, even with modern search and access tools. This applies to data from universal screening programs in the United States of America [National Newborn Screening and Genetics Resource Center (NNSGRC), 2011], the United Kingdom [Streetly et al., 2009], and the French overseas territories [Bardakdjian-Michau et al., 2009]. Although data of high quality are collected, we have been unable to access reliable aggregated data for these countries. At a time when several developing countries, particularly in Africa, are moving toward the implementation of such programs [Ohene-Frempong et al., 2008], the development of guidelines and tools allowing optimal use of high volumes of data is going to be a major challenge.

Furthermore, existing online resources face various challenges [Galperin and Fernandez-Suarez, 2012]. Several of the resources reviewed here have stopped being maintained and updated, or are simply inaccessible, usually due to a lack of funding. We believe that, by improving collaborations between the different groups leading these resources and developing a better integration of the various databases, it will be possible in the long term to reduce the likelihood of such events, as well as to minimize the costs associated with their development and maintenance. In the short term, we hope to make the resource presented here sustainable, to provide regular updates and to add further data on the thalassemias and HbE [Colah et al., 2010; Olivieri et al., 2008], as we assemble data and develop tailored mapping models for these disorders.

## Conclusions

We need epidemiological evidence to assess disease burden and to target interventions in an evidence-based manner, and this is as true for genetic diseases as it is for infectious diseases. It is crucial to gather data on the distribution and prevalence of these disorders and we present a new resource created by the MAP. A concerted effort from public health bodies, researchers and clinicians involved in malaria-related red cell disorders is necessary to scale up this work, and better integration of online resources would make it possible for public health workers and data modelers to find a comprehensive suite of information in one place. We strongly advocate the inclusion of geographical information in databases of genetic disorders to allow the assessment of the distribution of these disorders and highlight areas where their burden is highest.
